# Effect of Standardized Cranberry Extract on the Activity and Expression of Selected Biotransformation Enzymes in Rat Liver and Intestine

**DOI:** 10.3390/molecules190914948

**Published:** 2014-09-18

**Authors:** Hana Bártíková, Iva Boušová, Pavla Jedličková, Kateřina Lněničková, Lenka Skálová, Barbora Szotáková

**Affiliations:** Department of Biochemical Sciences, Charles University in Prague, Faculty of Pharmacy, Heyrovského 1203, Hradec Králové 50005, Czech Republic; E-Mails: Hana.Bartikova@faf.cuni.cz (H.B.); Iva.Bousova@faf.cuni.cz (I.B.); jedlicp@faf.cuni.cz (P.J.); lnenickk@faf.cuni.cz (K.L.); skaloval@faf.cuni.cz (L.S.)

**Keywords:** proanthocyanidin, anthocyanin, cytochrome P450, drug-metabolizing enzymes, enzyme activity

## Abstract

The use of dietary supplements containing cranberry extract is a common way to prevent urinary tract infections. As consumption of these supplements containing a mixture of concentrated anthocyanins and proanthocyanidins has increased, interest in their possible interactions with drug-metabolizing enzymes has grown. In this *in vivo* study, rats were treated with a standardized cranberry extract (CystiCran^®^) obtained from *Vaccinium macrocarpon* in two dosage schemes (14 days, 0.5 mg of proanthocyanidins/kg/day; 1 day, 1.5 mg of proanthocyanidins/kg/day). The aim of this study was to evaluate the effect of anthocyanins and proanthocyanidins contained in this extract on the activity and expression of intestinal and hepatic biotransformation enzymes: cytochrome P450 (CYP1A1, CYP1A2, CYP2B and CYP3A), carbonyl reductase 1 (CBR1), glutathione-S-transferase (GST) and UDP-glucuronosyl transferase (UGT). Administration of cranberry extract led to moderate increases in the activities of hepatic CYP3A (by 34%), CYP1A1 (by 38%), UGT (by 40%), CBR1 (by 17%) and GST (by 13%), while activities of these enzymes in the small intestine were unchanged. No changes in the relative amounts of these proteins were found. Taken together, the interactions of cranberry extract with simultaneously administered drugs seem not to be serious.

## 1. Introduction

Cranberry (*Vaccinium macrocarpon*, Ericaceae), a native plant of North America, is among the top selling dietary supplements around the world. The juice as well as dietary supplements derived from this berry exert various beneficial effects on human health, including prevention and treatment of urinary tract infections, anti-cancer and antioxidant activities [1,2]. However, the positive effect of cranberry juice in the prevention of urinary tract infections has recently been disputed [3,4]. The biological activities of cranberry can be attributed to a diverse group of phytochemicals, including phenylpropanoids (such as flavonoids and resveratrol), phenolic acids and isoprenoids such as lutein and ursolic acid [5,6]. Cranberries also represent a very good source of vitamin C [7]. Flavonoid constituents found in cranberry fruit belong primarily to three classes: proanthocyanidins, anthocyanins and flavonols [8]. Proanthocyanidins (also known as polyflavan-3-ols) found in cranberry fruits are primarily dimers, trimers and larger oligomers of (‒)-epicatechin, containing two types of linkages between epicatechin units: the more common C4β → C8 (B-type) linkage and a less common A-type linkage featuring both C4β → C8 and C2β → O7 interflavanoid bonds [9]. Among anthocyanins, glycosides of cyanidin, peonidin and petunidin prevail. The most abundant flavonol aglycone is quercetin, followed by myricetin and kaempferol. Cranberry fruits contain up to 91.5 mg of anthocyanins, 40 mg of flavonols and 180 mg of proanthocyanidins (with degrees of polymerization ≤ 10) per 100 g of ripe fruit [5].

Consumption of cranberry juice and/or various dietary supplements containing cranberries have always been considered safe, however, several studies have suggested that cranberry juice is capable of interacting with drug-metabolizing enzymes and thus may elicit clinically relevant interactions with certain drugs. For instance, cranberry juice inhibited the activity of cytochrome P450 3A (CYP3A) *in vitro* in human liver and rat intestinal microsomes [10]. Moreover, in rats, cranberry juice was as effective as grapefruit juice in enhancing the systemic exposure of nifedipine, the calcium channel antagonist and CYP3A substrate. In both cases, the area under the curve (AUC) of nifedipine was increased by 60% compared to saline [10]. Indeed, enteric, but not hepatic, CYP3A-mediated first-pass metabolism of midazolam was inhibited by cranberry juice in healthy volunteers [11]. In contrast, no interaction has been found in the human study involving CYP3A substrate cyclosporine and cranberry juice [12]. Long-term treatment with three cranberry extracts had no effect on glutathione S-transferase (GST) activity in rats [13]. Besides, weak inhibitory activity of cranberry extract towards UDP-glucuronosyl transferase 1A9 (UGT1A9) in human liver microsomes was reported [14]. Individual anthocyanidins were able to significantly inhibit human and rat carbonyl reductase (CBR) and UGT *in vitro* [15].

The abovementioned data show that information about the effect of cranberry extract/juice on drug-metabolizing enzymes has been inconsistent and insufficient. The inconsistence of the results from various studies is mainly due to the use of different experimental conditions. There are differences not only in the model systems, but also in the administered substances (undefined cranberry juice, defined cranberry extract, individual anthocyanidins, *etc*.), and dosage schemes (various doses, various treatment durations, *etc*.). Moreover, most of the studies have focused only on selected enzymes in one organ (mostly liver) and a comprehensive view has been lacking.

Therefore, the present *in vivo* study was designed to test the effect of cranberry extract (in two different dosage schemes) on the activity and expression of a panel of drug-metabolizing enzymes (a total of seven) in rats. In addition to the hepatic enzymes most studied in the literature, intestinal enzymes were also included in this project to obtain more complete information. With the aim to increase reproducibility and repeatability of our experiments, CystiCran^®^, a patented and standardized cranberry extract, was used in this study.

## 2. Results and Discussion

Cranberry standardized extract CystiCran^®^ (CC, Decas Botanical Synergies, Carver, MA, USA) contains 1.6 mg of anthocyanidins and 36 mg of proanthocyanidins in one tablet, mainly those comprising A-type linkages, which have been associated with preventing adhesion of uropathogenic bacteria to uroepithelial cells [16]. In the present study, male rats were orally treated with CC to simulate consumption of cranberry juice and/or dietary supplements containing cranberry extract. Cranberry juice as well as dietary supplements are usually taken orally, therefore *p.o.* administration of CC (by gastric gavage) was chosen. The two dosage schemes employed represented two possible situations: regular long-term consumption of dietary supplement as a prevention of urinary tract infection (14 days, 0.5 mg of proanthocyanidins/kg/day, *i.e.*, 1 tablet of CC/day in human therapy) and short-term overdose by dietary supplement (1 day, 1.5 mg of proanthocyanidins/kg/day, *i.e.*, 3 tablets of CC/day in human therapy). In order to obtain more comprehensive information about the impact of cranberry extract on drug-metabolizing enzymes, their activities and expressions were studied not only in liver, but also in small intestine. Moreover, anthocyanins and proanthocyanidins are polyphenols with low absorption [17], and thus their systemic bioavailability is low, but intestinal mucosa exposure is high.

Proanthocyanidins are stable during gastric transit. While degradation of polymeric proanthocyanidins to the corresponding monomers is negligible in the gastrointestinal tract *in vivo*, proanthocyanidin oligomers with a degree of polymerization lower than 5 are absorbable [18]. Upon absorption, all dietary polyphenols in the human body are subject to substantial transformation catalyzed by drug-metabolizing enzymes [19]. Unlike extensive phase II metabolism of absorbed monomers, which are glucuronidated, sulfated and methylated [19], phase II metabolism of dimers appeared to be limited because glucuronidated or sulfated metabolites of dimers were not detected in biological fluids after intestinal perfusion in rats [18]. Nevertheless, the majority of proanthocyanidins reach the colon intact and are degraded into phenylvalerolactones and phenolic acids by colon microbiota. These microbial metabolites may contribute to the health-promoting properties of proanthocyanidins *in vivo* [18].

### 2.1. Effect of CystiCran^®^ on the Activities of Phase I Biotransformation Enzymes

The phase I of drug metabolism includes oxidation, reduction or hydrolytic reactions, which lead to introduction or uncovering of functional groups (e.g., –OH, –COOH, –SH, –O– or –NH_2_ group) resulting in new chemical entities with different physico-chemical and biological properties. Reactions carried out by phase I enzymes usually transform active drugd into their inactive metabolite(s), but in certain cases, phase I biotransformation causes bioactivation of xenobiotics. Enzymes responsible for phase I biotransformation of drugs and other xenobiotics are abundantly present in the liver, gastrointestinal tract, lungs and kidneys. Many of them are inducible by various xenobiotics. Induction of drug metabolism may arise as a consequence of increased synthesis, decreased degradation, activation of enzymes or a combination of these three processes, although it should be emphasized that the majority of enzymes are induced at the transcriptional activation level. Thus, enzyme induction takes place only after its prolonged exposure to the xenobiotic [20]. Cytochromes P450 (CYP), a superfamily of the most important drug-metabolizing enzymes, are involved in the metabolism of about 75% of all drugs. These enzymes utilize one molecule of oxygen and produce oxidized substrates and a molecule of water [21].

**Figure 1:**
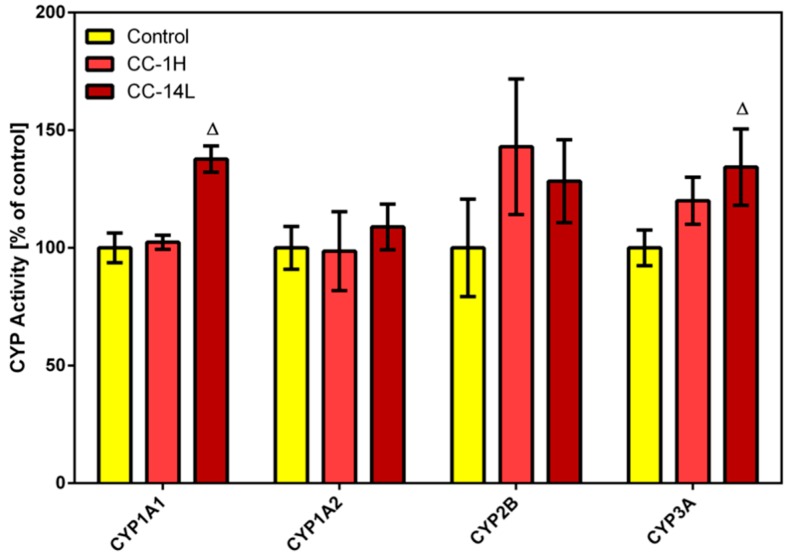
Effect of cranberry extract administered in two dosage schemes on the specific activities of cytochrome P450 isoforms 1A1, 1A2, 2B and 3A in rat liver microsomes. Specific activities were determined fluorimetrically using alkoxyresorufines as substrates. Data represent the mean ± SD of three independent experiments. The triangle indicates a significant difference from the control (*p* < 0.05).

In this study, specific activities of four CYP isoforms (*i.e.*, CYP1A1, 1A2, CYP2B, and CYP3A) were assessed in microsomal fractions of rat liver (Figure 1) and small intestine using alkoxyresorufins as substrates. Long-term treatment with low dose of cranberry extract (CC-14L) caused increase in the catalytic activity of hepatic CYP1A1 and CYP3A by 38% and 34%, respectively. Non-significant elevation in hepatic CYP2B activity was observed in CC-14L as well as in CC-1H groups. In rat small intestine, no specific activities of selected CYP isoforms were detected either in control or in CC-treated rats.

While in a study using healthy volunteers cranberry juice/extract caused inhibition of CYP3A [11], our results showed mild increases in CYP1A1 and CYP3A activities. This discrepancy may be caused by the different experimental models. Moreover, cranberry extract also contains other constituents such as the flavonoid quercetin and the natural stilbene resveratrol, whose *in vivo* inductive effects on CYP3A activity were described [22,23]. As the CYP3A subfamily is involved in the oxidative biotransformation of numerous drugs, modulation of CYP3A activity can have profound clinical consequences, but only with drugs that have narrow therapeutic windows [24]. Induction of CYP1A1/2 is also considered to be undesirable as these isoforms carry out bioactivation of polycyclic aromatic hydrocarbons (e.g., benzo[a]pyrene), recognized carcinogens in humans and rodents. Fortunately, induction of CYP1A1/2 and CYP3A by cranberry extract was only mild and thus serious consequences could be excluded.

**Figure 2:**
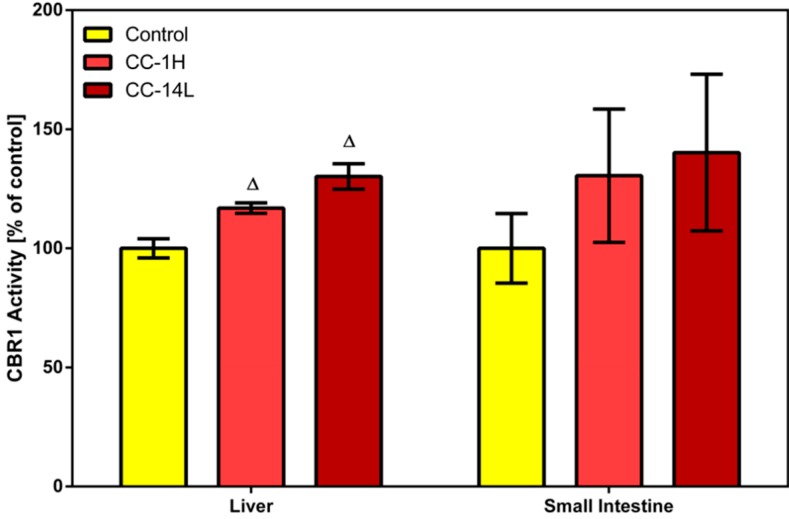
Effect of cranberry extract administered in two dosage schemes on the specific activity of carbonyl reductase 1 in the cytosolic fractions of rat liver and small intestine. Specific activities were determined fluorimetrically using menadione as a substrate. Data represent the mean ± SD of three independent experiments. Triangles indicate a significant difference from the control (*p* < 0.05).

Phase I reducing reactions of xenobiotics are often catalyzed by carbonyl reductase 1, a member of the short-chain dehydrogenases/reductases family, that reduces a wide variety of carbonyl compounds including quinones, prostaglandins, menadione, and various xenobiotics [24]. In our study, specific activity of CBR1 was measured in cytosolic fractions of liver and small intestinal mucosa homogenates using menadione as a specific substrate. Treatment of rats with CystiCran^®^ with both dosage schemes caused an elevation in CBR1 activities in the liver and small intestine, although only the changes found in the liver were statistically significant. Activity of hepatic CBR1 was increased by 17% and 30% in the CC-1H and CC-14L groups compared to untreated control, respectively (Figure 2).

CBRs, particularly isoform CBR1, are leading biotransformation enzymes catalyzing deactivation of carbonyl-bearing drugs, including cytostatics. Therefore, regulation of their activity has been intensively studied and by this way, several flavonoids (e.g., quercetin, quercitrin and rutin) and anthocyanidins (e.g., delphinidin, cyanidin and malvidin) have been recognized as *in vitro* inhibitors of CBR1 [15,25]. To our knowledge, no *in vivo* studies covering this topic have been performed yet. However, these compounds may act as inducers of CBR1 or may not to influence its activity *in vivo*. Their inductive/inhibitory effect is dependent also on the concentration used [26,27].

### 2.2. Effect of CystiCran^®^ on the Activities of Phase II Conjugating Enzymes

In phase II, xenobiotics or their phase I metabolites undergo conjugation reactions with endogenous compounds such as glutathione or UDP-glucuronic acid. Conjugation usually introduces hydrophilic ionizable functional groups onto the molecule of a xenobiotic, thus making it more polar and facilitating its renal excretion. Almost all phase II biotransformation reactions lead to detoxification/deactivation of the parent xenobiotic or metabolites formed in phase I. Glucuronidation, catalyzed by UGT, is the most common conjugation pathway in humans. Typical UGT substrates are xenobiotic or eobiotic alcohols, phenols or carboxylic acids. On the other hand, electrophilic compounds are mostly conjugated with glutathione through the action of GSTs [28].

**Figure 3:**
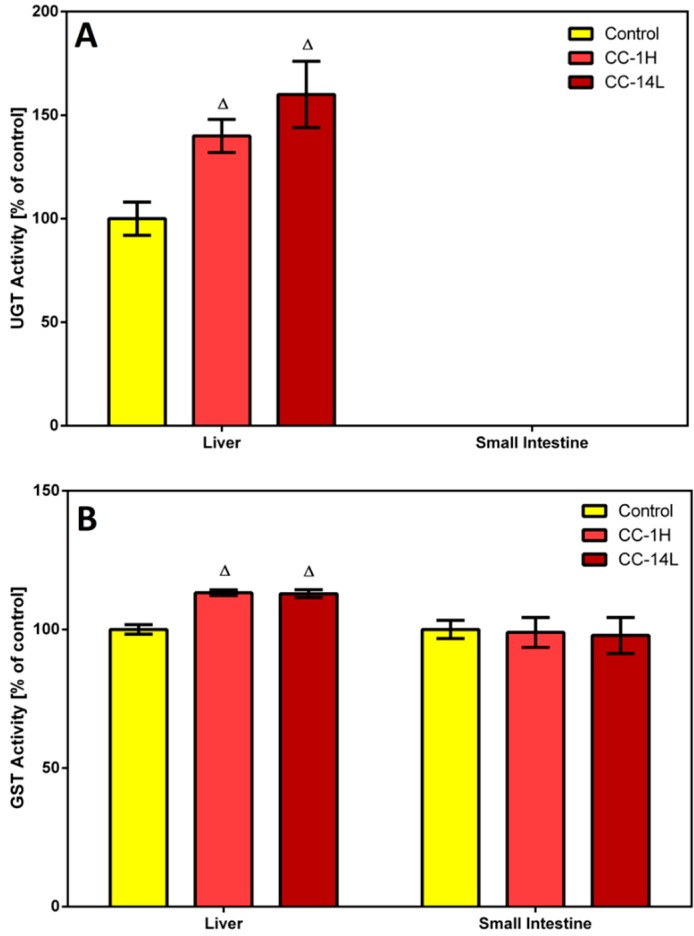
Effect of cranberry extract administered in two dosage schemes on the specific activities of UDP-glucuronosyl transferase in the microsomal fractions of rat liver and small intestine (**A**) and glutathione S-transferase in the cytosolic fractions of rat liver and small intestine (**B**). Specific activities were determined spectrophotometrically using *p*-nitrophenol and CDNB as UGT and GST substrates, respectively. Data represent the mean ± SD of three independent experiments. Triangles indicate a significant difference from the control (*p* < 0.05).

In this study, homogenates of rat liver and small intestine were analyzed for UGT and GST activities. Specific activity of UGT was tested in microsomal fractions using *p*-nitrophenol as a substrate. In the liver, UGT activity was significantly increased in CC-treated rats compared to the control group (Figure 3A).

On the other hand, specific UGT activity in the small intestine was at the detection limit, therefore no changes in this activity were detected. Catalytic activity of GST was assessed in cytosolic fractions by measuring its conjugation activity with the universal substrate 1-chloro-2,4-dinitrobenzene (CDNB). GST activity was elevated by 13% in the liver of both CC-treated groups compared to the control group, while an insignificant decrease in GST activity was observed in the small intestine of rats treated with CC (Figure 3B).

Several constituents found in cranberry may be responsible for increased GST and UGT activities in rat liver. Consumption of various fruits and plants, which are rich sources of anthocyanidins as well as proanthocyanidins, was shown to elevate UGT and GST activities in rat liver [29,30]. Observed induction of UGT activity may be caused also by quercetin, whose content in cranberry is estimated to be in the range of 83–121 mg/kg (*i.e.*, about 50 µg per 0.5 mg cranberry supplement capsule) [31]. This assumption was confirmed by the study of van der Logt *et al.* [32], in which significant induction of *p*-nitrophenol glucuronidation was found in rat liver and small intestine after 14-day intake of quercetin. Another constituent capable of UGT and GST activities induction is the stilbene resveratrol. This compound caused 1.8-fold and 1.3-fold increases of UGT and GST activities in the liver of male rats upon 28-day intake of resveratrol, respectively [33]. Moreover, 28-day administration of resveratrol to healthy volunteers led to induction of GSTP protein levels and UGT1A1 activity in individuals with low baseline enzyme level/activity, while overall GST and UGT1A1 activities were minimally affected by this intervention [22]. Induction of GST activity by cranberry extract can be considered protective, because increased levels/activity of GST, especially in the gastrointestinal tract, can prevent organisms against harmful electrophiles [26].

Certainly, it is necessary to take into consideration that the observed effects of cranberry extract may not be ascribed to only one chemical constituent as it is a complex mixture of various phytonutrients. Individual components of this mixture may interact with each other and the resulting biological activity of the cranberry extract is therefore dependent on their synergistic/antagonistic behavior. Moreover, biological effects of cranberry juice/extract are concentration-dependent. Thus, its inhibitory activity observed *in vitro* need not to be found also *in vivo* because the concentrations of active compounds in organism is influenced by their bioavailability. While high concentrations of bioactive compounds directly influence the activities of the studied enzymes in the *in vitro* experiments, the activity as well as expression of these enzymes are affected by low concentrations of these compounds in the *in vivo* study.

### 2.3. Expression of Biotransformation Enzymes

In our study, the activities of drug-metabolizing enzymes were assessed primarily as the activities which are more important than protein and mRNA levels from a pharmacological/toxicological point of view. When elevated activity of a certain enzyme was found, the corresponding protein was quantified using an immunoblotting technique. The amounts of liver CYP1A1, CYP3A4, and UGT1A proteins were detected in the microsomal fractions of control as well as CC-treated rats, while levels of hepatic CBR1 and GSTP proteins were quantified in the cytosolic fractions. Calnexin and β-actin were used as loading controls in microsomal and cytosolic fraction, respectively. Protein quantification did not revealed any significant changes in the proteins amount after CC treatment (Table 1).

**Table 1:** Effect of cranberry extract administered in two dosage schemes on the protein level of CYP1A1, CYP3A, UGT (microsomal fractions), CBR1 and GST (cytosolic fractions) in rat liver. Protein expressions were detected by immunoblotting using specific antibodies and normalized to the amount of loading controls calnexin (microsomal fractions) and β-actin (cytosolic fractions). Data represent the mean ± SD of three independent experiments.

Enzyme	Mean (%) ± SD		
	Control	CC-1H	CC-14L
CYP1A1	100.0 ± 12.3	89.8 ± 17.1	100.2 ± 9.3
CYP3A4	100.0 ± 9.6	108.2 ± 12.7	116.0 ± 10.4
CBR1	100.0 ± 14.2	106.3 ± 8.6	111.5 ± 15.9
UGT	100.0 ± 12.1	108.2 ± 5.9	100.7 ± 11.0
GST alpha	100.0 ± 7.9	104.5 ± 8.3	112.8 ± 7.6

Although the protein quantification was not in full agreement with the activities of biotransformation enzymes, similar trends were found in several studied enzymes (e.g., CYP3A4, CBR1 and GST alpha). Some discrepancies may be explained by differences in enzyme activity assessment and performance of immunoblotting. In the case of UGT and GST, enzyme activity was assessed using universal substrates covering most of the isoforms, while immunoblotting was performed using antibody specific for one isoform (GST alpha) or one enzyme family (UGT1A). Therefore, observed changes in enzyme activity may be caused by different isoform(s) (e.g., GST pi). Moreover, modulation of various biotransformation enzymes by cranberry extract may occur at several levels (transcriptional, posttranscriptional *etc.*) [34].

## 3. Experimental Section

### 3.1. Chemicals and Reagents

Benzyloxyresorufin, 7-ethoxyresorufin, 7-methoxyresorufin, 7-pentoxyresorufin, menadione, 1-chloro-2,4-dinitrobenzene (CDNB), 4-nitrophenol (NP), reduced glutathione (GSH), UDP-glucuronic acid, NADPH, and chemicals used for realization of electrophoresis were products of Sigma-Aldrich (Prague, Czech Republic). Precision Plus molecular weight standard and non-fat dry milk were purchased from Bio-Rad (Bio-Rad Laboratories, Hercules, CA, USA). For immunoblotting, rabbit polyclonal anti-UGT antibody (Cell Signaling, Leiden, The Netherlands), rabbit polyclonal anti-GST alpha antibody, rabbit monoclonal anti-CBR1 antibody, rabbit polyclonal anti-calnexin antibody, rabbit polyclonal anti-beta actin antibody (Abcam, Cambridge, UK), rabbit polyclonal anti-CYP1A1 antibody (Novus Biologicals, Cambridge, UK), rabbit polyclonal anti-CYP 3A4 antibody (Sigma-Aldrich), bovine anti-rabbit IgG antibody conjugated with horseradish peroxidase and chemiluminescence kit (Santa Cruz Biotechnology, Santa Cruz, CA, USA) were used. All other chemicals used were of HPLC or analytical grade.

### 3.2. Laboratory Animals

Male Wistar rats were obtained from Meditox (Konarovice, Czech Republic). They were housed in air-conditioned animal quarters with a 12 h light/dark cycle. Food (a standard rat chow diet) and water were provided *ad libitum*. The animal protocols used in this work were evaluated and approved by the Ethic Committee of the Ministry of Education, Youth and Sports (Protocol 20363/2011-30). They are in accordance with the Guide for the Care and Use of Laboratory Animals (Protection of Animals from Cruelty Act No. 246/92, Czech Republic). At 12 weeks of age, rats were randomly divided into three groups of four. Rats of the first group (CC-14L) were orally administered with therapeutic dose of CystiCran^®^ once daily for a period of 2 weeks; three times higher dose of CystiCran^®^ was administered at once by gastric gavage to the second group of rats (CC-1H; 24 h before the end of experiment), and the third group represents untreated controls. At the end of experiment, animals were sacrificed by decapitation. Livers and small intestines were removed immediately, the intestinal contents were washed out with cold 0.9% saline solution and mucosa was scraped. Both tissues were stored at −80 °C until preparation of subcellular fractions.

### 3.3. Preparation of Microsomal and Cytosolic Fractions

Frozen liver or mucosa from small intestine were thawed at room temperature up to 15 min and processed to microsomal and cytosolic fractions. The subcellular fractions were isolated by differential centrifugation of the tissue homogenate [35] and stored at −80 °C. Protein concentrations were assayed using the bicinchoninic acid (BCA) assay according to manufacturer's instructions (Sigma-Aldrich).

### 3.4. Enzyme Assays

Enzyme activities were assayed in the cytosolic and microsomal fractions from homogenates of liver and small intestinal mucosa of control and treated rats. The enzyme assays (each performed in 4–8 replicates) were repeated three times. The amount of organic solvents in the final reaction mixtures did not exceed 1% (v/v).

The activities of several CYP isoforms were assessed. The activities of 7-ethoxyresorufin *O*-dealkylase (EROD; specific for CYP1A1), 7-methoxyresorufin *O*-dealkylase (MROD; CYP1A2), 7-pentoxyresorufin *O*-dealkylase (PROD; CYP2B) and 7-benzyloxyresorufin *O*-dearylase (BROD; CYP3A) were measured at 37 °C using fluorimetric determination of arising resorufin [36]. Each substrate dissolved in dimethylsulphoxide (DMSO) was added at a final concentration of 5 µM. Assays were conducted at the excitation/emission wavelengths of 530/585 nm using luminescence spectrophotometer LS50B (Perkin-Elmer, Cambridge, UK).

Carbonyl reductase 1 activity was measured in the cytosolic fractions using menadione as a substrate [37]. Consumption of NADPH was determined at excitation/emission wavelength of 380/460 nm using a Perkin Elmer LS50B luminescence spectrophotometer at 37 °C.

The cytosolic glutathione S-transferase activities were assessed by standard colorimetric assay using CDNB as an electrophilic substrate [38], which was adapted for measurement in 96-well plates. The absorbance of rising product *S*-(2,4-dinitrophenyl)glutathione was detected at 340 nm by Tecan Infinite M200 multimode microplate reader (Tecan Group, Männedorf, Switzerland).

The microsomal UDP-glucuronosyltransferase activities towards *p*-nitrophenol were assayed as described by Mizuma *et al.* [39]. Absorbance of unconjugated *p*-nitrophenol was measured at 405 nm by the Tecan Infinite M200.

### 3.5. Western Blotting

Microsomal proteins of rat liver were separated by SDS-PAGE (10% stacking gel) [40] and subsequently transferred onto nitrocellulose membranes (0.45 μm) using Trans-Blot^®^ TurboTM Transfer System (Bio-Rad, Hercules, CA, USA). Protein concentrations were determined using the BCA protein assay (Sigma-Aldrich). The membranes were blocked in 5% non-fat dry milk/TBS-Tween-20 for 2 h. Immunodetection of biotransformation enzymes was performed using corresponding primary antibodies (described in the Chemicals and Reagents section). The bands were visualized with respective horseradish peroxidase-conjugated secondary antibodies using the chemiluminescence kit according to manufacturer's instructions. Calnexin and β-actin served as the loading controls for microsomal and cytosolic fraction, respectively. Intensity of bands was evaluated using a C-DiGit^TM^ Blot Scanner (Li-Cor, Lincoln, NE, USA).

### 3.6. Statistical Analysis

All calculations were done using Microsoft Excel and GraphPad Prism 6. All values were expressed as mean ± SD. One-way Anova was used for the statistical evaluation of differences between control and treated groups, and differences were considered as significant when *p* < 0.05.

## 4. Conclusions

In conclusion, *in vivo* administration of standardized cranberry extract in both studied dosage schemes caused only mild changes of some activities of drug-metabolizing enzymes in rat liver, while those in small intestine were not affected. Interestingly, long-term consumption of regular dose has more pronounced effects on drug-metabolizing enzymes’ activities than short-term overdose by cranberry extract. Nevertheless, consumption of cranberry juice/extract in reasonable amounts seems to be safe and serious supplement–drug interactions do not seem probable.
